# Mitochondria in cancer

**DOI:** 10.15698/cst2020.06.221

**Published:** 2020-05-11

**Authors:** Debora Grasso, Luca X. Zampieri, Tânia Capelôa, Justine A. Van de Velde, Pierre Sonveaux

**Affiliations:** 1 Pole of Pharmacology & Therapeutics, Institut de Recherche Expérimentale et Clinique (IREC), Université catholique de Louvain (UCLouvain), Brussels, Belgium.

**Keywords:** tumor metabolism, tricarboxylic acid (TCA) cycle, oxidative phosphorylation (OXPHOS), reactive oxygen species (ROS), apoptosis, mitophagy, mitochondrial biogenesis

## Abstract

The rediscovery and reinterpretation of the Warburg effect in the year 2000 occulted for almost a decade the key functions exerted by mitochondria in cancer cells. Until recent times, the scientific community indeed focused on constitutive glycolysis as a hallmark of cancer cells, which it is not, largely ignoring the contribution of mitochondria to the malignancy of oxidative and glycolytic cancer cells, being Warburgian or merely adapted to hypoxia. In this review, we highlight that mitochondria are not only powerhouses in some cancer cells, but also dynamic regulators of life, death, proliferation, motion and stemness in other types of cancer cells. Similar to the cells that host them, mitochondria are capable to adapt to tumoral conditions, and probably to evolve to ‘oncogenic mitochondria' capable of transferring malignant capacities to recipient cells. In the wider quest of metabolic modulators of cancer, treatments have already been identified targeting mitochondria in cancer cells, but the field is still in infancy.

## INTRODUCTION

The vital role of mitochondria in eukaryotic cells has been demonstrated over a hundred years ago by Otto Warburg, who was the first to perform mitochondrial respiration experiments [1]. In healthy replicative eukaryotic cells, mitochondria regulate important cellular processes, such as proliferation, death, metabolic adaptation and Ca^2+^ homeostasis. Mitochondria are also the site of important reactions, including fatty acid oxidation (FAO), the tricarboxylic acid (TCA) cycle, oxidative phosphorylation (OXPHOS), the first step of gluconeogenesis, ketogenesis, heme biosynthesis and Fe/S cluster formation [2]. They contain DNA that can vary with evolution, mutate or be partially deleted. Given the numerous functions of mitochondria, it is not surprising that mitochondrial dysfunctions participate in a series of diseases, including cancer. In this review, we focus on mitochondrial functions and their contribution to carcinogenesis and cancer progression.

## MITOCHONDRIA PARTICIPATE IN CANCER DEVELOPMENT

There are at least five mechanisms by which mitochondria may be involved in the development of the malignant phenotype over the metabolic reprogramming of cancer cells. First, it is widely demonstrated that a large number of diseases are associated with DNA mutations that affect mitochondria, mainly due to alterations of subunits of the electron transport chain (ETC) [3]. For example, subsets of hepatocellular carcinomas and prostate cancers have been associated with a mutation in the D-loop region of Complex I, and some neurological cancers harbor mutations of succinate dehydrogenase (SDH; Complex II) [4–6]. Second, oxidative stress due to reactive oxygen species (ROS) is the most important stimulus for cancer generation and progression towards malignancy [7]. ROS are mainly produced by mitochondria that release superoxide as a byproduct of oxidative respiration [8]. Mitochondrial ROS (mtROS) can be generated either in the TCA cycle or in the ETC [9]. Due to their high reactivity, ROS act as toxic species for cellular macromolecules [10] and, at low concentrations, as intracellular signaling agents regulating metabolic pathways [11, 12]. Increased levels of ROS are often found in cancer cells due to increased metabolic activities and altered antioxidant capacities [13]. Third, mitochondria are directly involved in the regulation of cell death, including but not limited to apoptosis and necrosis [14, 15]. To induce apoptosis, B-cell lymphoma-2 (Bcl-2) family member proteins interact with mitochondria as they bind to the voltage-dependent anion channel (VDAC) to accelerate its opening and the release of cytochrome *c* [16]. Thereby, these proteins act as oncogenic or oncosuppressive triggers, participating in cancer progression and therapeutic resistance [17, 18]. One of them, myeloid leukemia cell differentiation protein-1 (MCL-1), an anti-apoptotic member of the Bcl-2 family, is frequently overexpressed in human cancer and associated with tumor aggressiveness [19]. MCL-1 and Bcl-xL have been found in different mitochondrial subcompartments. They exert their anti-apoptotic activities by antagonizing the pro-apoptotic members of the Bcl-2 family when located at the outer mitochondrial membrane (OMM) [20], and, when located in the mitochondrial matrix, by regulating mitochondrial homeostasis and bioenergetics by preserving the integrity of the inner mitochondrial membrane (IMM) and promoting the assembly of ATP-synthase oligomers at the ETC [17]. Mitochondria also control necroptosis, a regulated form of necrosis that needs mtROS generation and depends on mitochondrial permeability transition [21]. Fourth, metabolic reprogramming also concerns several mutations in genes encoding TCA cycle enzymes, which promote malignant transformation [22]. Indeed, some TCA cycle intermediates, such as fumarate, succinate, aspartate and *D*-2-hydroxyglutarate (2HG, a *de novo* metabolite resulting from mutations of isocitrate dehydrogenases (IDHs)), have important pro-carcinogenic effects when accumulating in cells following genetic mutations and/or cancer-associated modifications of protein expression [23]. Fifth, a distinctive feature of all tumors is sustained cellular proliferation resulting from multiple molecular alterations. One of these alterations is the prevention of telomere erosion by constitutive telomerase expression that ensures the maintenance of telomere length [24]. It has been shown that telomerase reverse transcriptase (TERT) shuttles from the nucleus to mitochondria upon oxidative stress, preserving mitochondrial functions and decreasing oxidative stress, thus protecting mitochondrial DNA (mtDNA) and nuclear DNA (nDNA) from oxidative damage to avoid apoptosis [25, 26]. TERT was also found to accumulate in the mitochondria of brain cells in mice upon dietary restriction and rapamycin treatment [27].

## MITOCHONDRIA ARE NOT ONLY THE POWERHOUSES OF THE CELL

Despite the fact that mitochondria are well recognized to actively participate in cancer progression, their precise roles in the clinical outcome of cancer patients remain elusive. The interest of scientists for mitochondria has increased over the last 50 years, with discoveries on the impact that these organelles have in multiple vital processes in eukaryotic cells [28].

Mitochondria are tubular organelles of ∼0.5 to ∼3 µm in length that undergo a continuous remodeling of their network by fusion and fission events [29]. Textbooks first describe mitochondria as the main site of energy production of cells, and, indeed, mitochondria are a major site of production of ATP and macromolecules. The reactions of the TCA cycle take place in the mitochondrial matrix. Together with CO_2_ and protons, they generate reducing equivalents (NADH and FADH2) and precursors for the synthesis of lipids, carbohydrates, proteins and nucleotides. Equivalent-reducing electrons fuel the ETC to generate an electrochemical gradient that is required both for ATP production and for the active transport of selective metabolites, such as pyruvate and ATP, across the IMM [30].

**Figure 1:**
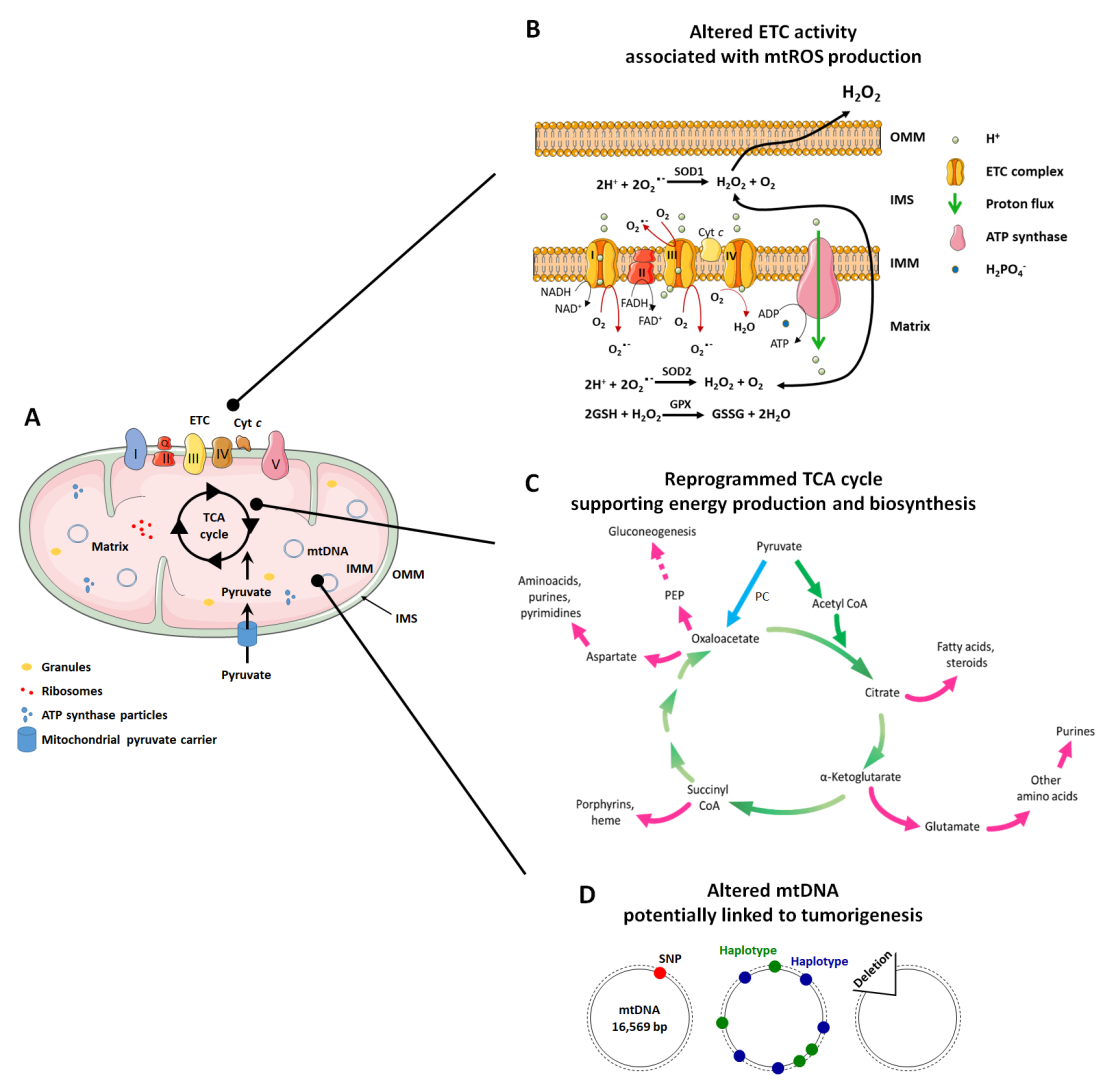
FIGURE 1: Cancer is associated with alterations of mitochondrial functions. **(A)** Mitochondria in normal and in cancer cells are composed of three compartments. They are separated from the cell cytosol by an outer membrane (OMM), an intermembrane space (IMS), and an inner membrane (IMM) that forms invaginations called "crests". The IMM delimitates the mitochondrial matrix, a gelatinous material containing mitochondrial DNA (mtDNA), granules, ribosomes and ATP synthase particles. The mitochondrial matrix hosts the tricarboxylic acid (TCA) cycle, while the IMM hosts the electron transport chain (ETC). **(B)** In highly metabolically active or hypoxic cancer cells much more than in normal cells under normal conditions, electrons escape during mitochondrial electron transport at Complexes I and III generate superoxide (O_2_^-^) from oxygen (O_2_) in both the IMS and the matrix. O_2_^-^ is immediately dismutated to H_2_O_2_ either spontaneously or under the catalysis of superoxide dismutases SOD1 (in the IMS) or SOD2 (in the matrix). In the matrix, H_2_O_2_ can be neutralized by glutathione (GSH). It can also signal to the cytosol. **(C)** In cancer cells, the TCA cycle not only serve to produce reducing equivalents to fuel the ETC (green arrows), but also to generate biosynthetic intermediates that are necessary for cell proliferation (pink arrows). The most important anaplerotic reaction produces oxaloacetate directly from pyruvate, and is catalyzed by pyruvate carboxylase (PC) (blue arrow). Oxaloacetate can further be converted to phosphoenolpyruvate (PEP) by PEP carboxykinase (PC), contributing to gluconeogenesis. **(D)** Mitochondrial DNA (mtDNA) variations, including single nucleotide polymorphisms (SNPs), maternally inherited haplotypes and deletions have been studied for their association with cancer. Among these, only large mtDNA deletions seem to be associated with malignancies. Cyt *c -* cytochrome *c*; Gpx - glutathione peroxidase; Q - coenzyme Q10.

In addition to this important role, mitochondria are implicated in many other functions related to mitochondrial dynamics and architecture, which influence some of the most important cellular activities. The mitochondrial structure (**Figure 1A**) is intrinsically connected to mitochondrial functions (ATP production, cell cycle control, programmed cell death control, proliferation and cell signaling) [31]. Mitochondria are indeed composed of two membranes, the OMM and the IMM that delimitate an intermembrane space (IMS) and the mitochondrial matrix inside the organelle. The OMM can be considered as a platform for exchange and signaling, as it is the site where proteins phos-phorylate substrates and regulate the immune response after viral infection trough activation of mitochondrial antiviral signaling (MAVS) proteins [32, 33]. The IMM is less permeable and is the site where ETC complexes are located for ATP production and superoxide generation [34]. The matrix is the site of mitochondrial mtDNA replication, transcription and macromolecule biosynthesis, where amphibolic reactions of the TCA cycle take place [35]. The investigation of mitochondrial mechanisms that control metabolic alterations and mitochondrial morphology has produced evidence that, in pathologies like cancer, they can be attractive targets for therapy.

## THE MITOCHONDRION: A COMPARTMENTALIZED ORGANELLE

### The OMM: a platform for signaling

A main function of mitochondria is to ensure that the cell does not undertake processes for which mitochondria are not suitable, thus avoiding a discrepancy between cellular metabolic requirements and the mitochondrial capacity. There are different ways by which mitochondria can communicate with the cell. One of them is through the OMM, which can be compared to a signaling platform. The OMM connects mitochondria to other organelles within the cell, such as the endoplasmic reticulum (ER) and lysosomes, and to the plasma membrane [36, 37]. Thanks to VDAC that forms pores composed of different subunits, the OMM is permeable to small ions and proteins [38]. VDAC carries nucleotides, ions and metabolites between the cytosol and the IMS [38], and acts as an intracellular signaling platform for the modulation of metabolism and the control of cell death [39, 40]. With respect to metabolic regulation, VDAC also acts as platform for the anchoring of hexokinase 2 (HK2), the embryonic version of the first enzyme of glycolysis, to the OMM to facilitate the use of ATP by HK2 in cancer cells [41]. Because glucose phosphorylation by HK2 traps glucose-6-phosphate within cells, this binding implies that VDAC is involved in the regulation of both glycolysis and mitochondrial respiration. With respect to cell death, the binding of Bcl-2 family members (Bax, Bak, Bok, Bad, Bid or Bim) to VDAC leads to the formation of a pore that results in the release of cytochrome *c* [16, 42], a small hemoprotein free to spread among the different mitochondrial compartments [43]. Cytochrome *c*, besides being a component of the ETC and, therefore, being necessary for the production of ATP at the ETC, also induces caspase-dependent cell death in response to pro-apoptotic stimuli [44].

Mitochondria also exert a control on the innate immune system. The OMM is indeed the site of phosphorylation of a wide range of proteins. For example, protein kinase A (PKA), a tetramer composed of two subunits that bind cAMP and two catalytic subunits, is a mitochondrial resident [45]. Mechanistically, a-kinase-anchoring proteins (AKAPs) are found in the OMM where they allow the binding of the PKA catalytic subunits to the organelle membrane, facilitating PKA localization for protein phosphorylation [32, 46]. When cAMP binds to PKA, the two catalytic subunits are dissociated, becoming active and phosphorylating a wide range of target proteins that have the arginine-arginine-X-serine motif exposed, such as splicing factors SRSF1, SRSF2 and SRSF9 [47]. The OMM is also home to antiviral signaling regulators that activate the immune response thanks to MAVS, as recently reviewed in details by Mohanti A *et al.* [48].

OMM signaling is only one of the ways mitochondria communicate with the rest of the cell. Indeed, mitochondria play a vital role in other important signaling pathways. First, they house the production of acetyl-CoA and S-adenosylmethionine that both regulate epigenetics by controlling signal transduction of DNA and histones through acetylation and methylation, respectively [49, 50]. Second, mtROS production and release is a response to cellular stress and a signaling factor to notably activate transcription factors hypoxia-inducible factors (HIFs), nuclear factor erythroid-derived 2-like 2 (Nrf2) and downstream gene expression [51]. Third, mitochondria control Ca^2+^ homeostasis. Ca^2+^ acts as a signaling molecule between mitochondria and the ER through contact sites termed ‘mitochondrial associated membranes' (MAMs) [52]. It is one of the most important signals that these organelles use for communication [53]. Ca^2+^ acts as a bidirectional signaling molecule, as mitochondrial Ca^2+^ uptake regulates mitochondrial metabolism, while mitochondrial Ca^2+^ release modulates apoptosis. Indeed, an increased Ca^2+^ concentration inside mitochondria activates several TCA cycle enzymes [54] and stimulates the production of cAMP [55], which in turn increases ATP production, allowing metabolic adaptation. Conversely, Ca^2+^ is a signal for programmed cell death, with high levels of Ca^2+^ inducing the opening of mitochondrial permeability transition pores (MPTPs), triggering the release of cytochrome *c* and initiating apoptosis [56]. Ca^2+^ released from ER-mitochondria contact sites can also activate apoptosis through Bcl-2 family members upon a fine regulation of Ca^2+^ homeostasis [57]. Furthermore, changes of ATP production by mitochondria act as a signal that is transmitted to the cytosol in the form of AMP: AMP activates energy sensor AMP kinase (AMPK), thus decreasing anabolic cell functions in favor of the catabolic reactions used for ATP production [58].

Overall, mitochondrial signaling is a dynamic and complex process that affects most cellular functions. By understanding these processes, it may be possible to more effectively treat diseases like cancer.

### The IMM: the ATP factory

Compared to the OMM, the IMM does not contain porins and is a highly impermeable barrier to ions and molecules that require specific membrane transport proteins for bidirectional exchanges [59]. The IMM has an electrochemical membrane potential of about 180 mV that regulates their passage. It is also more extensive than the OMM, as it is organized in invaginations called ‘mitochondrial cristae' [60] to allow the arrangement of ETC complexes. The number of invaginations depends on the energy demand of the tissue. In muscles, for example, mitochondria are particularly rich in cristae [61].

The IMM further differs from the OMM by its high protein content, with a protein-to-lipid ratio of 80:20 in the IMM and of 50:50 in the OMM [59]. This high protein content is represented by all the complexes responsible for OXPHOS, as well as by transport proteins and proteins that regulate fusion and fission. The respiratory chain consists of a series of multi-enzymatic complexes and hydro- and liposoluble compounds capable of transferring electrons sequentially through the complexes towards the final acceptor, O_2_. This electron flow creates a motor force that transfers protons from the mitochondrial matrix (their site of production) to the IMS against their concentration gradient, thus generating an electrochemical gradient across the IMM. The various complexes are arranged in increasing order of reduction potentials, in such a way that transported electrons pass from a higher energy state to a lower energy state with consequent energy release, which is used, in part, for ATP synthesis. Another part of the energy produced is used for thermoregulation [62]

The respiratory chain is composed of five complexes (three of which are proton pumps) and two carriers that act as co-substrates and electron transporters (**Figure 1A and 1B**):

**Complex I - NADH dehydrogenase, also called coenzyme Q (CoQ) reductase.** Complex I is composed of 45 subunits. Among them, 14 have catalytic activities and are called ‘essential subunits', of which seven are hydrophobic (ND1, ND2, ND3, ND4, ND4L, ND5 and ND6) and encoded by the mitochondrial genome, and the other seven are hydrophilic [63]. The remaining subunits are called ‘non-essential subunits'. They are important for the assembly and stability of Complex I and are encoded by the nuclear genome [64]. Complex I receives two hydrogen atoms from NADH, which is oxidized, releasing two electrons and reducing flavin mononucleotide (FMN) to FMNH_2_. The latter transfers the electrons to the second carrier of the ETC, CoQ, through its Fe/S centers. The energy obtained from the passage of electrons is used by Complex I to transport four protons from the mitochondrial matrix to the IMS, which represents a flow of two protons for each NADH consumed [65]. Complex I dysfunctions have been associated with cancer, as a reduced activity of the complex has been observed in renal oncocytomas [66, 67] and thyroid adenomas [68].

**CoQ.** CoQ is a lipophilic ubiquinone carrier embedded in the IMM lipid bilayer. It is able to separate the protons (which are released in the mitochondrial matrix) from the electrons provided by FMNH_2_ [69].

**Complex II - SDH.** Complex II is composed of four nuclear encoded subunits, with two hydrophilic catalytic subunits, SDHA/SDH1 and SDHB/SDH2, and two hydrophobic subunits, SDHC/SDH3 and SDHD/SDH4 [63]. It contains a heme b group and two CoQ-binding sites. It is also part of the TCA cycle. Complex II contributes to electron transfer, but there is no proton pumping towards the IMS. The two electrons produced during the oxidation of succinate to fumarate are directly transferred to CoQ [70]. The reduction activity of Complex II has been shown to be associated with human cancer in renal carcinoma [71] and breast cancer [72], where Complex II activity is lower compared to the corresponding normal tissues.

**Complex III - Cytochrome bc1 complex, also called Cytochrome *c* reductase.** Complex III is a symmetrical dimer, and each subunit is composed of three catalytic cores (MT-CYB, CYC1 and UQCRFS1) and seven supernumerary subunits [63]. This complex receives electrons from CoQ and passes them to cytochrome *c*; then, it carries four protons towards the IMS [73]. A higher than physiological activity of Complex III has been detected in breast cancer [72, 74].

**Cytochrome *c*.** Cytochrome *c* is a hydrophilic heme protein located at the outer surface of the IMM. It transfers electrons between Complexes III and IV [75].

**Complex IV - Cytochrome *c* oxidase.** Complex IV is composed of 13 or 14 subunits, and it is the only OXPHOS complex containing tissue-specific and developmentally regulated isoforms [63]. This complex transfers four electrons (provided by four molecules of cytochrome *c*) directly to O_2_ (provided by the blood), reducing it to two molecules of H_2_O, which consumes four protons (taken from the mitochondrial matrix) [76]. A reduced activity of Complex IV has been observed in cancer. In renal carcinomas, Complex IV expression was found to be 5-fold lower compared with healthy kidney tissues [71].

**Complex V - ATP synthase.** Complex V is composed of two distinct domains. The F1 domain is extrinsic to the IMM and is found in the matrix, while the Fo domain is intrinsic to the IMM. F1 is composed of nine subunits, while Fo has two subunits [63]. ATP synthase is the complex where ATP is produced from the substrates ADP + H_2_PO_4_^-^ + H^+^ in a reaction at the end of the OXPHOS process. While electrons traveling across the ETC are finally transferred to O_2_ at Complex IV, most of the protons that were transferred from to mitochondrial matrix to the IMS return through the ATP synthase complex (a channel protein), nullifying the electro-chemical gradient. The energy released by the return of the protons according to their electrochemical potential is used in the form of mechanical energy to allow the functioning of ATP synthase [77].

Recent data have suggested that ETC complexes are organized in supermolecular structures called ‘supercomplexes' [78]. These structures appear to be organized in different ways and to adopt stoichiometry [79]. For example, Complex I is frequently bound with a dimer of Complex III and with Complex IV [80]. This type of supercomplex is called ‘respirasome' because it contains all the complexes responsible for the transfer of electrons from NADH down to O_2_. It has also been shown that Complex I is always associated with supercomplexes, while Complexes III and IV also exist as free oligomeric enzymes [80]. This suggests that the assembly of the complexes in a single structure is necessary for the stabilization and the correct functioning of Complex I in the respiratory chain [81]. Indeed, several studies have shown that loss of Complexes III and IV also causes the loss of Complex I [82, 83].

ETC activity is associated with cancer in several ways. A growing amount of experimental evidence indicates that OXPHOS affects the production of ATP more significantly in cancer cells than in normal cells [84, 85]. In particular, OXPHOS is used for a massive production of ATP by invasive and metastatic cells, as well as by circulating cancer cells [86]. Further evidence shows that cancer stem cells (CSCs) utilize OXPHOS as a preferred form of energy metabolism, and in general they display higher rates of oxygen consumption, ROS production and an overall increase in mitochondrial functions compared to non-stem cancer cells [87]. Moreover, mtROS promote V-Ki-ras2 Kirsten rat sarcoma viral oncogene homolog (KRAS)-induced anchorage-independent growth [88].

As demonstrated by Porporato *et al.* [89], an ETC overload can increase the migration, invasion, clonogenicity and metastatic potential of cancer cells through a mitochondrial superoxide-dependent mechanism that activates the transforming growth factor β (TGFβ) pathway at the level of src kinase. Despite mitochondrial respiration can be perceived as an intriguing target for cancer treatment, it has been amply demonstrated that tumors are metabolically heterogeneous and influenced by different substrates and factors from the tumor environment [90]. Not only does metabolism differ among the various cancer types, but it also differs between subpopulations of cancer cells within the same tumor [91, 92]. Cellular subpopulations with different metabolic phenotypes (elevated OXPHOS or dysfunctional glycolysis) have been identified in melanoma [93], lymphomas [94], pancreatic [95] and breast [96] tumors.

### The mitochondrial matrix: the fulcrum of metabolism

The mitochondrial matrix is the inner mitochondrial space delimited by the IMM. It mostly hosts mtDNA, ribosomes, enzymes, small organic molecules, nucleotide cofactors and inorganic ions [97]. The mitochondrial matrix is a viscous space due to its high protein content with respect to the IMS, and it has a higher pH than the IMS (7.8 in the matrix; 7.0-7.4 in the space) [98]. It is the site of numerous enzymatic reactions, including those of the TCA cycle, anaplerotic and cataplerotic reactions, the urea cycle (at least in part), transamination reactions and part of ETC reactions (Complex II).

#### The TCA cycle

The TCA cycle (also known as Kreb's cycle or citric acid cycle, **Figure 1C**) is the most important process that takes place in the mitochondrial matrix. It is composed of a series of chemical reactions capable of processing two-carbon units from carbohydrates, amino acids and fatty acids in acetyl-CoA to generate GTP and the reducing equivalents (NADH and FADH2) that fuel the mitochondrial ETC to generate ATP [97]. Acetyl-CoA is oxidized in a cyclic metabolic pathway to CO_2_, with a net production of one CoA-SH, two CO_2_, three NADH, one FADH2, one GTP/ATP and three H^+^ for each molecule of acetyl-CoA consumed.

The first reaction of the cycle is the condensation of acetyl-CoA with oxaloacetate by citrate synthase (CS) to form citrate. Citrate is then converted to its isomer, isocitrate, by mitochondrial aconitase (ACO2) and subsequently decarboxylated to α-ketoglutarate (α-KG) by mitochondrial IDH. In this reaction, a CO_2_ molecule is released and a NAD^+^ molecule is reduced to NADH + H^+^. A second decarboxylation occurs when α-KG is converted to succinyl-CoA by α-KG dehydrogenase, which also produces NADH + H^+^. The next reactions serves to regenerate oxaloacetate through (1) oxidation of succinyl-CoA in succinate by succinate-CoA synthetase, which produces GTP; (2) oxidation of succinate to fumarate by SDH with the production of a molecule of FADH2; (3) hydration of fumarate to malate by fumarate hydratase (FH); and, finally, (4) oxidation of malate by malate dehydrogenase to regenerate oxaloacetate, which produces NADH + H^+^ [97].

The TCA cycle is a focal point of cellular metabolism with a central importance for both energy production and biosynthesis. Therefore, to retain the homeostasis of cellular metabolism, a balance between intermediate production and consumption must be maintained [99]. Anaplerotic reactions are a series of enzymatic reactions that produce metabolic intermediates aimed to replenish the TCA cycle, whereas cataplerotic reactions are biosynthetic reactions that use TCA cycle intermediates as substrates for macromolecule synthesis [99] (**Figure 1C**). The TCA cycle, in fact, not only produces NADH and FADH2 useful for ATP synthesis at the ETC, but it also provides molecules useful for other metabolic pathways, such as gluconeogenesis and the synthesis of fatty acids and nucleotides [35].

The most important anaplerotic reaction produces oxaloacetate directly in mitochondria starting from pyruvate, a reaction which is catalyzed by pyruvate carboxylase (PC) [100]. This reaction is allosterically regulated by acetyl-CoA and aspartate, which signal a deficiency in oxaloacetate [101]. Other anaplerotic reactions produce different intermediates of the TCA cycle, including α-KG produced from glutamate by glutamate-dehydrogenase, succinyl-CoA produced by FAO, and oxaloacetate produced from aspartate by aspartate transaminase [100].

When the production of intermediates of the TCA cycle is sufficient for its correct functioning, cataplerotic reactions intervene to balance metabolite concentrations [100]. Hence, citrate can be exported to the cytosol and converted to acetyl-CoA by ATP citrate lyase, initiating fatty acids biosynthesis. In a reversible reaction, α-KG with aspartate produce glutamate and oxaloacetate by the activity of aspartate transaminase, participating in purine synthesis [99]. Oxaloacetate can also be converted to phosphoenolpyruvate (PEP) by PEP carboxykinase (PEPCK), contributing to gluconeogenesis [99].

#### Alterations of the TCA cycle in cancer

Mutations in genes encoding TCA cycle enzymes and the abnormal accumulation of TCA cycle intermediates can promote carcinogenesis [102, 103]. The main enzymes that were found to be altered in cancer are SDH, FH, IDH, CS and ACO2, which by itself highlights an extensive area open to investigation. Mitochondrial abnormalities induce metabolic reprogramming with cells increasingly relying on glycolysis, which further supports the tumorigenic process [104].

The first studies on the implication of *SDH* mutations in cancer showed that patients with hereditary paragangliomas and pheochromocytomas, two rare neuroendocrine neoplasms, displayed inactivating mutations of subunits of SDH enzymes [105, 106]. As a consequence, succinate accumulates in these tumors.

Inactivating mutations of *FH* predispose to hereditary leiomyomatosis, renal cancer and multiple cutaneous and uterine leiomyomas [107–109]. Indeed, upon accumulation, fumarate acts as an oncometabolite that inhibits α-KG-dependent dioxygenases involved in DNA and histone demethylation [110, 111]. It also promotes the epithelial to mesenchymal transition (EMT) by inhibiting ten eleven translocation (TET)-dependent DNA demethylation of a regulatory region of antimetastatic miR-200, leading to a decreased expression of miR-200 and E-cadherin, and an increased expression of Twist1 and vimentin, among other target genes [112]. Defects of FH further stimulate the nuclear translocation and activity of Nrf2, and the consequent transcription of antioxidant genes through antioxidant response elements (AREs). These genes comprise mitochondrial residents thioredoxin 2, thioredoxin reductase 2, peroxiredoxins and superoxide dismutase 2 (SOD2), as well as transporters and enzymes involved in glutathione (GSH) biosynthesis and redox recycling (see reference [113] for a recent review). Nrf2 activity is mitigated by Kelch-like ECH-associated protein 1 (KEAP1) that interacts with Nrf2 in the cytosol, targeting it for polyubiquitylation followed by proteasomal degradation [114], and by transcription modulators that heterodimerize with Nrf2 in the cell nucleus [115]. Conversely to its induction of antioxidant systems, fumarate can directly react with GSH to produce succinated GSH that mimics the GSH reductase substrate [116]. This causes NADPH consumption without antioxidant effects, thus increasing oxidative stress.

IDH presents three isoforms, with NADP^+^-dependent IDH1 found in the cytoplasm and in peroxisomes, and NADP^+^-dependent IDH2 and NAD^+^-dependent IDH3 found in the mitochondrial matrix. *IDH1* and *IDH2* have been reported to be mutated in 70% of grade II and III gliomas and glioblastomas [117, 118], as well as in angio-immunoblastic T-cell lymphomas [119], acute myeloid leukemia (AML) [120, 121] and other common cancer types, such as thyroid, colorectal and prostate cancers [122, 123]. *IDH1* and *IDH2* mutations decrease NADPH and GSH levels, leading to enhanced PI3K-AKT-mTOR signaling pathway activity and cancer cell migration [124, 125]. Several studies have shown that carcinogenesis and cancer progression are modulated by *IDH* mutations, and it has been reported that these mutations increase ROS levels in cancer cells [126, 127]. Both increased ROS levels and PI3-AKT pathway activation lead to an increased signaling activity in favor of cancer formation and progression [128, 129]. Interestingly, mutated *IDH*s most often gain the function of producing 2HG, the R enantiomer of α-KG [130]. 2HG is an oncometabolite that, when accumulated at a very high concentration, inhibits α-KG-dependent dioxygenases [131], such as histone lysine demethylases, leading to a hypermethylated state of DNA and histones [132]. It also activates HIF-1 by inhibiting HIF-1α degradation through prolylhydroxylases [133].

CS is an important enzyme often considered to be the rate-limiting enzyme of the TCA cycle [134]. Its loss causes a metabolic shift from an oxidative to a glycolytic metabolism and induces EMT, increasing tumor malignancy [135]. However, a high expression of CS has been observed in pancreatic, renal and ovarian cancers [136–138].

A decreased expression of ACO2 has been associated with gastric [139] and prostate [140] cancers. Interestingly, *FH*-deficient cells also have impaired aconitase activity because fumarate accumulating in these cells exerts a dose-dependent inhibition of ACO2 activity via succination of critical cysteine residues [141].

When inactivating mutations occur in TCA cycle enzymes, metabolic intermediates different than the direct substrates of mutated enzymes may accumulate, and they can act as oncometabolites [102, 103]. For example, citrate is an oncometabolite that, when accumulating, disrupts the equilibrium of the TCA cycle and promotes cancer development [142]. Indeed, high levels of citrate reduce the activity of pyruvate dehydrogenase, with consequent pyruvate accumulation [142]. In this situation, cells convert pyruvate to lactate with NAD^+^ regeneration, thus causing a metabolic shift towards glycolysis, a well-known phenomenon in cancer progression [143]. Furthermore, citrate is a substrate of acetyl-CoA carboxylase, by which acetyl-CoA and malonyl-CoA are produced as precursors for lipid and steroid synthesis [144]. On the one hand, lipids are the major structural components of biological membranes and play important functions in cell signaling promoting cell proliferation and transformation [145, 146]. On the other hand, acetyl-CoA derived from citrate is a mandatory substrate for histone acetylation, modulating chromatin structure, and, thus, gene transcription [147].

Histone acetylation consists of a dynamic and reversible acetyl group transfer process controlled by histone acetyltransferases (HATs) and histone deacetylases (HDACs) [147]. Acetylation of specific histones allows chromatin relaxation, which facilitates the transcription of important genes that regulate cell proliferation, cell cycle transition, differentiation and apoptosis, and have been linked to tumor development, influencing the cellular growth program of cancer cells [148]. The use of HDAC inhibitors has proven to be an effective therapy capable of reversing the transformed cellular phenotype, and several HDAC inhibitors have been approved by the FDA for cancer therapy [149, 150]. Of note, in addition to histones, oncoproteins, tumor suppressors and enzymes can be acetylated as well, which controls their activities. It is the case of HIFs, Myc, KRAS, p53, retinoblastoma protein pRb and PTEN, and of enzymes involved in glucose, fatty acid and glutamine metabolism [147].

#### Alterations of mtDNA in cancer

Both mtDNA mutations and deletions have been associated with cancer (**Figure 1D**).

Single nucleotide polymorphisms in mtDNA (mtSNPs) were tested for their potential correlation with an increased risk of developing cancer or with a more aggressive progression. While on the one hand a study correlated a decrease in mtDNA copy number with colorectal cancer malignancy [151], on the other hand a similar study excluded any correlation between mtDNA copy number and mtSNPs on cancer progression in colorectal cancer patients [152]. In this study, six mtSNPs (MitoT479C, MitoT491C, MitoT10035C, MitoA13781G, 10398 A/G and 16189 T/C) were analyzed in 536 patients and mtDNA copy number in 274 patients, comparing tumor and healthy tissues. A potential correlation between mtSNPs and the risk of prostate cancer has also been studied. Following the comparison of 350 mtSNPs between 4,086 cancer patients and 3,698 healthy subjects in a multiethnic cohort, the authors reported no association between mtSNPs and the risk of prostate cancer [153].

Ethnicities are characterized by different haplogroups, which can be defined as clusters of mtDNA sequence variations that are statistically inherited from the ancestral maternal mitochondrial genome [154]. For example, Europeans present nine major haplogroups (H, U, J, T, K, W, I, V and X) [155]. Differences in the mtDNA sequence can result in alterations of mitochondrial proteins, mostly components of the ETC, that influence their activity, ETC efficiency and ROS production. In oncology, haplogroups have been extensively studied, in particular regarding their possible correlation with the risk of developing specific cancer types. Analyses suggested that some haplogroups are indeed correlated with an increased risk for specific cancer types, whereas they simultaneously reduce the risk of other types of cancer [156]. For example, haplogroup K would statistically be protective against thyroid cancer in Southeastern Europeans, but would increase the risk of breast cancer in European Americans [157]. Another interesting study in a cohort of over 7,700 European individuals found no correlation between haplogroups and breast cancer risk factors, neither in mothers nor in their children [158]. A major difficulty to link haplogroups to cancer risks is due to the way results are analyzed. As a typical example, one study reported that haplogroup N is correlated with an increased risk of breast cancer [159], whereas another similar study reported that haplogroup U and haplogroup K are correlated with a decreased and an increased risk of breast cancer, respectively [160]. However, according to phylotree.org, haplogroup N contains haplogroup U, which in turn includes haplogroup K, making it complex to define what is the actual weight of these alterations on the risk of developing cancer, and making of the comparison of different studies a difficult task. From a molecular point of view, it is still unclear how a same set of mtDNA variations could simultaneously be pro- and anticarcinogenic.

Large mtDNA deletions usually result in severe alterations of the ETC and, consequently, in OXPHOS defficiency. Three main studies have analyzed the frequencies of these events in cancers. In the first study, a 4,977 bp mtDNA deletion has been analyzed in more than 1,600 samples of tumor and adjacent healthy tissues [161]. Results suggested that this particular deletion could be a cancer biomarker, especially in breast cancer. The second and third studies focused on the frequency of a 3,400 bp mtDNA deletion in prostate cancer [162, 163]. They reported that this deletion is highly frequent in prostate cancer and in the healthy tissue directly adjacent to the lesion, while it was absent in normal prostate epithelium. The 3,400 bp large mtDNA deletion was therefore proposed as a biomarker of prostate cancer.

Altogether, to the exception of large mtDNA deletions, the amount of contradictory studies regarding mtDNA variations and their correlations with cancers makes it impossible to conclude anything clinically relevant today. In addition to contradictions, the biological consequences of these alterations are poorly characterized at both molecular and functional levels. Consequently, targets cannot be easily identified and pharmacological approaches cannot be developed. The complete picture is even worse if one considers that the amount of studies with statistical or methodological fallacies, in particular regarding breast cancer, is so high that Salas A *et al.* wrote an entire article on that topic [164].

## MITOCHONDRIAL REACTIVE OXYGEN SPECIES

### Mitochondria as ROS producers

The term ‘ROS' refers to a class of highly reactive molecules containing oxygen and having a very short average half-life. ROS collectively design superoxide (O_2_^°-^), the hydroxyl radical (OH) and hydrogen peroxide (H_2_O_2_). Mitochondria are considered to be the most important producers of superoxide in cells [34].

Mitochondrial superoxide, the proximal ROS, is mainly produced at the ETC from the leakage of electrons at the ubiquinone-binding sites of Complex I (IQ site) and Complex III (IIIQo site) during electron transport [9] (**Figure 1B**). Superoxide production primarily occurs through the donation of a single electron from totally reduced or partially reduced electron carriers, such as semiquinone, to O_2_ [34]. The kinetics of O_2_^°-^ production depend on the carrier enzyme average time in a reduced form and on its concentration [34], highlighting that the concentration of enzymes responsible for O_2_^°-^ production tunes the functions that ROS play within cells. Another important variable is the O_2_ concentration in cells. Accordingly, altering experimentally the local O_2_ concentration can increase or decrease the rate of mitochondrial O_2_ consumption, leading to alterations of O_2_^°-^ production [34]. Being highly reactive, O_2_^°-^ is immediately dismutated to H_2_O_2_ either spontaneously or under the catalysis of mitochondrial SODs. SOD1 is present in the IMS and in the cytosol where it inactivates superoxide produced at ETC Complex III, whereas SOD2 is localized in the mitochondrial matrix where it inactivates superoxide produced at Complexes I and III [34] (**Figure 1B**).

In addition to ubiquinone-binding sites in ETC Complexes I and III, other ROS-producing sites have been identified in mitochondria: flavin in Complex I (IF site), the electron transferring flavoprotein Q oxidoreductase (ETFQOR) in FAO, glycerol 3-phosphate dehydrogenase, pyruvate dehydrogenase and 2-oxoglutarate dehydrogenase [165]. Although the mechanisms governing the functioning of each of those sites are not yet fully understood, studies have shown that all of them produce ROS in the mitochondrial matrix, while only the IIIQo site and glycerol 3-phosphate dehydrogenase can release O_2_^°-^ in the IMS [165]. In these two cases, once produced in the IMS, H_2_O_2_ can cross the OMM and exercise signaling activities in the cytosol [166] (see Section ‘Antioxidant defenses in mitochondria‘) for more details).

Strong evidence exists supporting the signaling role of mtROS as a mode of communication between mitochondria and cellular processes involved in the maintenance of homeostasis and adaptation to stress conditions [167]. mtROS play different signalization roles in cells by promoting cell survival or by enhancing cell death, and for mitochondrial recycling. First, mitochondrial H_2_O_2_ regulates posttranscriptional protein modification through oxidation of the thiol groups of cysteines, thus changing the activity of target proteins and, therefore, the final response [168]. For example, the reaction of H_2_O_2_ with phosphatases, such as mitogen-activated protein kinase phosphatase (MKP), inhibits their dephosphorylative activity, leading, e.g., to c-Jun N-terminal kinase (JNK) activation [169]. Second, an increased release of mtROS is an adaptive response of cells to hypoxia that activates HIFs [170]. ROS indeed oxidize the iron moiety of HIF prolylhydoxylases (PHDs), thus inhibiting the activity of these enzymes that normally initiate the process of HIF-1α degradation by catalyzing the hydroxylation of this HIF subunit [171]. ROS further oxidize vitamin C, which is a necessary co-factor for PHD recycling though Fe^3+^ reduction. Notably, HIF-1 is known to upregulate the expression of glycolytic enzymes and transporters to maintain sufficient ATP levels in cells [172] and to enhance the production of vascular endothelial growth factor (VEGF) for the simulation of angiogenesis [173]. Third, mtROS act as a signal for triggering autophagy, which exerts either prosurvival or pro-apoptotic effects [168]. During starvation, mtROS oxidize and inactivate cysteine protease Atg4, which triggers the lipidation of Atg8, an essential step in the process of autophagy, thus promoting autophagy and facilitating the recycling of intracellular molecules [174]. Interestingly, autophagy can further contribute to ROS accumulation due to selective catalase degradation [175]. Fourth, mtROS generate signaling responses at nuclear gene expression level by forming a perinuclear clustering of mitochondria, leading to an accumulation of ROS in the nucleus with subsequent alterations of gene transcription [176].

### Antioxidant defenses in mitochondria

**Figure 2:**
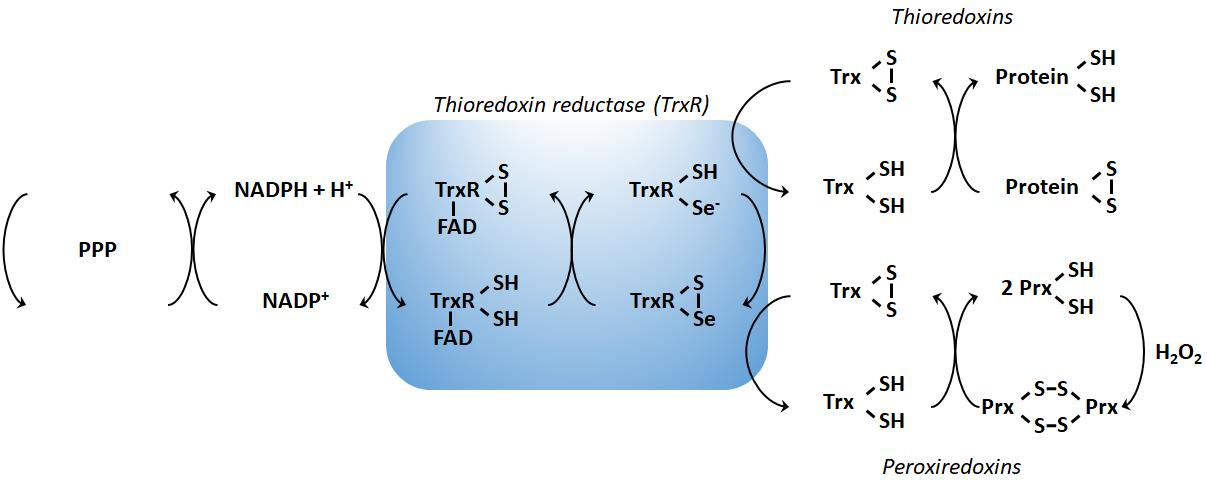
FIGURE 2: The arsenal of mitochondrial antioxidant defenses comprises the thioredoxin and peroxiredoxin pathways. The image depicts redox reactions catalyzed by the thioredoxin and peroxiredoxin systems, comprising thioredoxin reductases (TrxR, of which ThxR2 is expressed in mitochondria), thioredoxins (Trx), peroxiredoxins (Prx) and NADPH. The electron source is NADPH, which mostly originates from the pentose phosphate pathway (PPP). Oxidized thioredoxins (Trx-S2) are reduced by NADPH and selenoenzymes TrxRs. Electrons are sequentially transferred from NADPH to FAD, to the N-terminal redox active disulfide in one subunit of TrxR, and, finally, to the C-terminal active site of another subunit. Reduced thioredoxins (Trx-SH_2_) catalyze disulfide bond reduction in many proteins, including Prxs, thus ensuring oxidative damage repair in proteins as well as H_2_O_2_ detoxification.

In order to maintain the balance between ROS production and their harmful consequences, cells harbor specific defense mechanisms. They protect cells from detrimental ROS effects, i.e., damage to lipids, proteins and nucleic acids, and induction of double strand DNA breaks [177]. Superoxide produced in mitochondria is reduced to H_2_O_2_ by SOD1 (also known as cumin-zinc superoxide dismutase, CuZnSOD) in the IMS and in the cytosol, and by SOD2 (also known as manganese superoxide dismutase, MnSOD) in the mitochondrial matrix [34] (**Figure 1B**). Of note, SOD3 is also a member of the CuZnSOD family, but is extracellular [178]. After the conversion of superoxide to H_2_O_2_ by SODs, the subsequent conversion of two H_2_O_2_ to O_2_ + two H_2_O is catalyzed by peroxidases: catalase, located in peroxisomes, the thioredoxin system, and GSH peroxidase. The thioredoxin system coupled to the peroxiredoxins catalyzes the reduction of H_2_O_2_ to H_2_O in the presence of NADPH, and consists of thioredoxin peroxidases, thioredoxin reductases and the substrates thioredoxin an peroxiredoxin [179] (**Figure 2**). Mitochondria have their own specific thioredoxin reductase (ThxR2) for redox regulation and peroxiredoxin for the reduction of peroxides [180]. Finally, in the cytosol, GSH peroxidase catalyzes the conversion of H_2_O_2_ to H_2_O using GSH as a substrate, which is reduced to GSSG and then regenerated with the use of NADPH by GSH reductase [180]. Thus, GSH synthesis (via glutaminolysis and the serine pathway) and NADPH production actively support H_2_O_2_ inactivation [181].

## METABOLIC AND MITOCHONDRIAL CONTROL OF CELL DEATH

There is a clear connection between metabolism and cell death mediated by various signal transduction pathways. In cancer cells, p53 coordinates a common central pathway [182]. p53 is the most important pro-apoptotic protein and is mutated/inactivated in ∼50% of tumors [183]. However, while p53 has a central role in tumor suppression, it is also involved in the modulation of cancer metabolism. Two well-known targets of p53 that regulate cell metabolism are TP53-induced glycolysis and apoptosis regulator (TIGAR) [184] and synthesis of cytochrome oxidase 2 (SCO2), a cytochrome oxidase 2 (COX2) assembly protein [185]. While TIGAR decreases the glycolytic flux by dephosphorylating fructose-2,6-bisphosphate [184], SCO2 promotes ETC assembly and OXPHOS [186]. p53 is thus able to repress glycolysis and to promote OXPHOS and FAO [182].

Nutrient availability is commonly altered during tumor growth [187]. Abnormal tumor perfusion and, consequently, nutrient restriction, impact cell death. For instance, a common microenvironmental alteration in tumors is hypoxia, which can act as a signal for p53 activation and cell death induction [188]. In addition, the metabolic status of the cell acts as a signal for p53 induction: when cellular ATP levels decline, the resulting decrease in the ATP/AMP ratio activates AMPK, and AMPK phosphorylates/activates p53 on serine 15, thus initiating an AMPK-dependent cell-cycle arrest [189, 190].

AMPK acts in most cases as a tumor suppressor, not only by inducing a cell cycle arrest, but also by inhibiting the synthesis of most cellular macromolecules. AMPK activation indeed inhibits mammalian target of rapamycin complex 1 (mTORC1) by phosphorylating its upstream regulator tuberous sclerosis complex 2 (TSC2), thus inhibiting cell growth [191]. ATP levels and AMPK thereby provide an important connection between p53-mediated regulation of energy metabolism and programmed cell death [191]. Of note, adenosine signaling can further induce apoptosis by stimulating adenosine receptor A2B, which activates a caspase- and p53-upregulated modulator of apoptosis (PUMA)-dependent apoptotic response involving a downregulation of Bcl-2 expression [182].

Mitochondria are another important crossroad between metabolism and cell death. They control cell death through apoptosis (most notably by regulating the release of cytochrome *c* through VDAC and the mitochondrial transition pore), and some forms of necrosis [14, 192]. Mitochondria control the intrinsic apoptotic pathway through OMM permeability, which is tightly regulated by Bcl-2 proteins [193, 194]. A release of mitochondrial Ca^2+^ is critically involved in the initiation and effectuation of apoptotic cell death. In this context, AKT activation confers resistance to apoptosis by stimulating Bcl-2 protein expression [195], and the binding of HK2 to VDAC on the mitochondrial surface further represses apoptosis [196]. In addition to circumstances where mitochondrial integrity is altered, cell death is directly related to the metabolic activity of mitochondria. For example, inhibiting mitochondrial OXPHOS in renal cell carcinoma cells resistant to glucose starvation was reported to induce cell death under glucose deprivation [197].

Different from classical types of cell death, autophagy is considered to exert a dual function in cancer, as it is both a tumor suppressor and a protector of cancer cell survival [198]. Autophagy is indirectly modulated by metabolic enzymes. For example, lactate dehydrogenase-1 (LDH-1), catalyzing the conversion of lactate and NAD^+^ to pyruvate, NADH and H^+^, recently emerged as a modulator of autophagy [199]. LDH-1 indeed interacts with proton pump vacuolar (V)-ATPase at the surface of lysosomes, which it fuels with protons. Another example concerns glycolytic enzyme glyceraldehyde-3-phosphate dehydrogenase (GAPDH) [200]. During glucose deprivation, GAPDH is phosphorylated by AMPK and is translocated to the cell nucleus where it interacts with the NAD^+^-dependent deacetylase sirtuin 1 (Sirt1) [201]. Both AMPK-dependent phosphorylation and the nuclear translocation of GAPDH mediate rapid Sirt1 activation in the nucleus, leading to the transcriptional induction of the autophagic program [201].

Other sirtuins also exert their activities at the interface between cell metabolism and death. Sirt2 has dual effects on mitophagy. In normal metabolic conditions, it can translocate from the cytosol to mitochondria, where it forms a complex with heat shock protein 70 (Hsp70), preventing Hsp70 acetylation and, thereby, inhibiting mitophagy [202]. However, Sirt2 also exists as a heterodimer with forkhead O family protein 1 (FoxO1) in the cytoplasm of cancer cells: upon serum starvation or oxidative stress, the complex is disrupted, resulting in FoxO1 acetylation and FoxO1 activation of autophagy-related 7 (Atg7) through protein-protein interaction, which triggers autophagic cell death [203]. Comparatively, Sirt3 is a mitochondrial resident. Under hypoxia, it promotes mitophagy and prevents mtROS-induced apoptosis by facilitating the binding of Parkin to VDAC1 [204]. The exact mechanism underlying this effect is not well characterized. Sirt4-7 could also regulate mitophagy, but molecular details are lacking. Sirt4 would promote mitochondrial fusion, thereby limiting mitophagy [205], while Sirt5 would indirectly prevent mitophagy by limiting ammonia production [206]. The expression of both Sirt6 and Sirt7 was shown to be important to preserve functional autophagy in cancer cells [207, 208].

## MITOCHONDRIAL DYNAMICS

**Figure 3:**
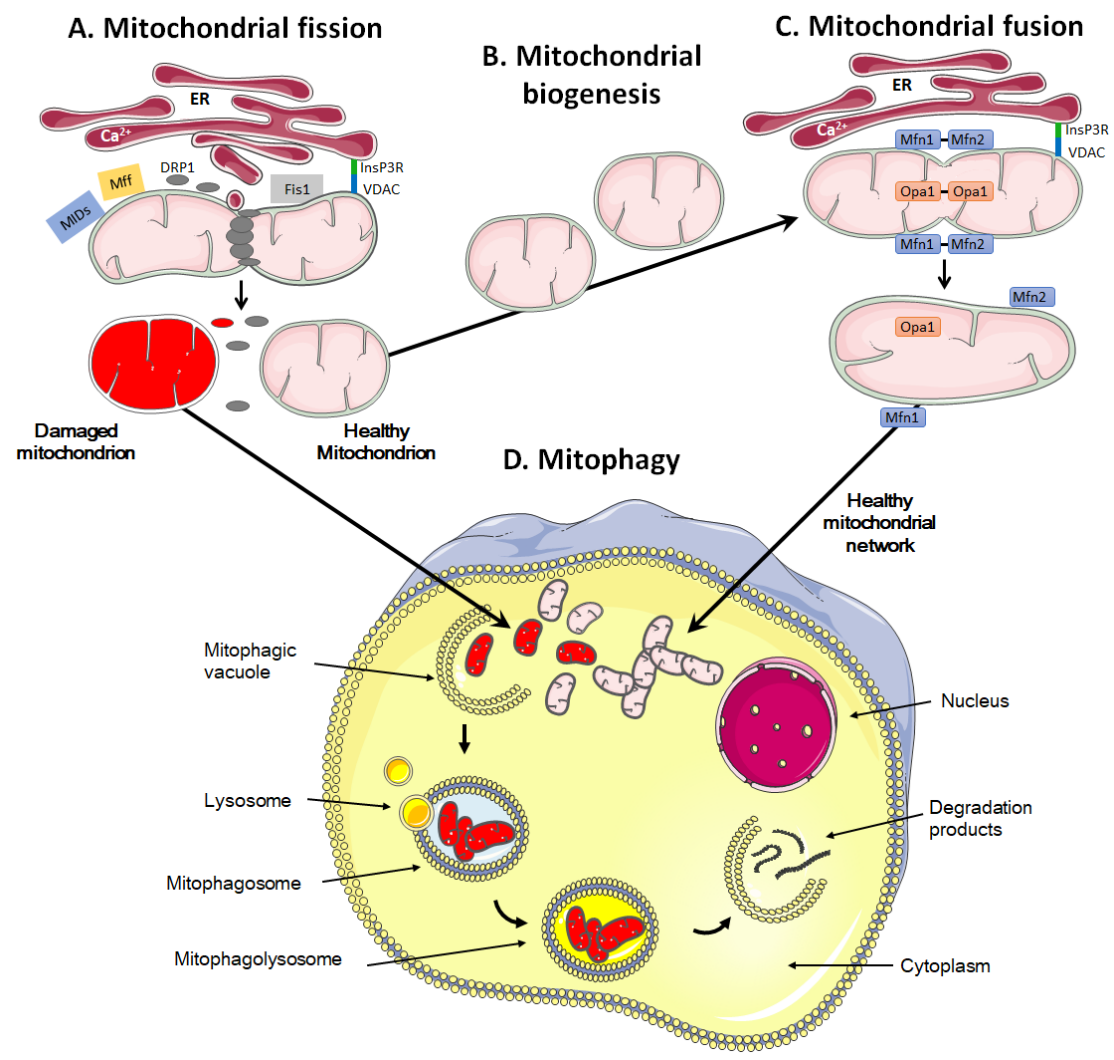
FIGURE 3: A high mitochondrial turnover rate is characteristic of many cancer cells. Mitochondrial quality control involves fission and mitophagy to eliminate defective mitochondria, whereas repopulation and functionalization depends on mitochondrial biogenesis and fusion. **(A)** During fission, the mitochondrion is marked and anchored by the endoplasmic reticulum (ER), notably through the binding of inositol 1,4,5-trisphosphate receptor (InsP3R) at the ER surface to voltage-dependent anion channel (VDAC) at the mitochondrial surface. This leads to the recruitment of dynamin-related protein 1 (DRP1), mitochondrial receptor protein 1 (Fis1), mitochondrial fission factor (Mff) and mitochondrial dynamic proteins (MIDs), allowing oligomerization and constriction to yield two daughter mitochondria. **(B)** During mitochondrial biogenesis, a mitochondrion self-replicates. **(C)** During fusion, mitofusins Mfn1 and Mfn2 are located on the outer mitochondrial membrane, allowing the exchange of calcium for signaling and creating antiparallel connections between two fusing mitochondria. Optic atrophy 1 (Opa1) together with Mnf1 participate in the fusion of the inner mitochondrial membrane. Fusion allows the formation of mitochondrial networks. **(D)** The mitophagic process consists in the engulfment of damaged mitochondria in a vacuole, called ‘mitophagic vacuole'. The subsequent fusion of the mitophagosome with lysosomes, forming a mitophagolysosome, allows the degradation of mitochondria in macromolecules that are delivered to the cytosol. Mitophagy can be non-selective or selective, using canonical and non-canonical pathways. It prevents the accumulation of damaged mitochondria that could harm or even kill the cell if apoptosis and/or the production of reactive oxygen species would derail.

Mitochondria are dynamic organelles that move through the cell, divide, fuse and undergo a regulated turnover through mitophagy [209, 210] (**Figure 3**). They can also be exchanged between cells. These mitochondrial dynamics reflect metabolic alterations.

### Mitochondrial fission and fusion

Mitochondrial fission is the process during which mitochondria divide into two or more independent structures, allowing the creation of new mitochondria. Conversely, mitochondrial fusion characterizes the merging of several neighboring mitochondria, which mixes the content of partially damaged mitochondria as a form of complementation [211]. Both processes require highly regulated mechanisms to allow the formation of new, functional organelles and to avoid a loss of intramitochondrial content.

During fission, mitochondria are divided into smaller pieces, an essential process to increase their number in dividing cells. Fission also promotes mitochondrial removal through mitophagy, facilitates the movement of mitochondria through the cytoskeletal network and regulates apoptosis and Ca^2+^ homeostasis [211–213]. The process is initiated by actin and the ER that first mark the site of division on the OMM [214, 215] (**Figure 3A**). Once the contact between ER and mitochondria is established through VDAC at the mitochondrial surface and the inositol 1,4,5-trisphosphate receptor InsP3R at the ER surface, the ER releases Ca^2+^ into the mitochondrion to trigger actin polymerization at the constriction site [216]. This provides a site for the recruitment and assembly of dynamin-related protein 1 (DRP1), a cytosolic GTPase that translocates to the OMM upon activation [217]. DRP1 then recruits membrane-anchored receptor protein Fis1 [218], tail-anchored mitochondrial fission factor (Mff) and anchored mitochondrial dynamic proteins (MiDs) [219]. Together, these proteins spirally surround the mitochondrion, constricting and breaking it in two pieces.

High DRP1 expression has been associated with different types of cancers, including glioblastomas, thyroid, lung and breast tumors [220–223], as well as with an increased metastatic potential of cancer cells [224, 225]. Interestingly, silencing DRP1 has been shown to reduce the metastatic capacity of breast cancer cells due to inhibition of lamellipodia formation, an important mechanism that drives cell migration [224]. DRP1 was also found to be overexpressed in squamous cell carcinomas (SCCs), and loss of DRP1 in this tumor type causes mitochondrial elongation with subsequent inhibition of cell proliferation and a G2 arrest [226]. Mechanistically, DRP1 expression was found to positively correlate with the expression of cell cycle genes that regulate mitosis in epithelial ovarian carcinoma (EOC) [227]. Elevated DRP1 expression promotes mitosis, thus supporting cell proliferation in the development of primary and relapsed EOC. DRP1 is also linked to cancer cell metabolism, since depletion of DRP1 in HeLa cells has been shown to decrease the activity of ETC complexes, mitochondrial respiration, mitochondrial membrane conductance and ATP synthesis [228, 229]. An intriguing link between mitochondrial fission and cancer comes from the functional connection between DRP1 and cellular stress, where DRP1 has been proposed as a transcriptional target of p53 [230] and where oncogenic RAS/MAPK signaling upregulates *DRP1* mRNA levels [223]. The multiple ways by which DRP1 is involved in cancer suggest that this protein plays important roles that could be independent of its principal role of ‘separating' mitochondria.

Numerous studies have addressed the metabolism of CSCs (see references [231, 232] for reviews). While their metabolic activities vary across tumor types, mitochondrial fission has been singled out to be important for stemness maintenance [233]. Accordingly, silencing DRP1 or its pharmacological inhibition in brain tumor-initiating cells reduced their tumorigenicity and triggered apoptosis [220]. Similarly, the pharmacological inhibition of DRP1 by mdivi-1 not only caused a defect in tumor sphere formation by breast CSCs, but also inhibited their migration and survival [234]. In another recent example, Civenni *et al.* [235] observed that depleting Mff in prostate CSCs rapidly exhausted their tumorigenic potential, which was associated with the induction of CSC senescence. Interestingly, the high rate of mitochondrial fission in CSCs has been proposed to allow mother stem cells to keep an intact mitochondrial content, whereas daughter cells committed to differentiate would inherit a pool of intact and deficient mitochondria [236]. This asymmetric segregation would participate in the perpetuation of cancer stemness despite frequent cell divisions [233].

During fusion, for example following mitochondrial replication (also known as mitochondrial biogenesis, **Figure 3B**), the OMM and the IMM merge, and the contents of the IMS and mitochondrial matrix are mixed (**Figure 3C**). Molecularly, fusion is controlled by three dynamin family GTPases, mitofusin (Mfn) 1 and 2 in the OMM and optic atrophy protein 1 (Opa1) in the IMM [237, 238]. Opa1 displays two long isoforms (L-Opa1) and three short isoforms (S-Opa1) [239]. The fusion of the OMMs is for the most part synchronized with the fusion of the IMMs. Fusion proteins are regulated through ubiquitination of OMM proteins and proteolytic cleavage of IMM proteins [240]. When two mitochondria are in close proximity, Mfn1 and Mfn2 start to dimerize with homo- and heterotypic interactions, which creates antiparallel connections between mitochondria, allowing the fusion of the OMM through mixing the lipid bilayers [241]. Further interaction of the two fusing mitochondria with the ER is believed to facilitate the process, as Mfn2 has also been found in the ER where it promotes the interaction between mitochondria and ER and allows the exchange of Ca^2+^ for signaling [242]. Fusion of IMMs is then ensured by Opa1 together with Mnf1 [243].

In cancer cells, mitochondrial fusion appears to have the opposite effect than fission in terms of tumor growth, metastatic capacity and metabolic activities. Indeed, lower levels of Mfn1/2 have been found in mouse medulloblastoma cancer cells compared to non-transformed cells [244], and inhibition of DRP1 by mitochondrial division inhibitor-1 (Mdivi-1) stimulated fusion and initiated mtDNA replication [245]. Moreover, Mfn2 overexpression reduced lung cancer growth [222], as well as the migration and invasion of breast cancer cell lines [224]. Hyperfused mitochondria are found during the G1/S phase of the cell cycle that is associated with a greater oxidative capacity and higher ATP production [245]. Interestingly, Mfn2 gene therapy, which involves the insertion of genetic material (DNA) into the cells to restore Mfn2 gene expression, has been reported to reduce the proliferation of A549 human lung cancer cells by promoting apoptosis [222].

Fusion and fission also allow a subcellular specialization of mitochondria [246]. Mitochondria indeed distribute subcellularly depending on where their metabolic function is required (local demand of ATP, Ca^2+^ buffering and other functions). For example, at the axonal level in neurons, mitochondria appear fragmented because they move along the cytoskeleton (interacting with desmin, vimentin and tubulin), while at the dendritic level in the region of synapses, they appear elongated because in this region the demand/consumption of ATP is higher for pumping back the ions derived from synaptic vesicles [247]. Fission facilitates the distribution of mitochondria within the cell, and apoptosis through the release of cytochrome *c* [248]. Conversely, fusion benefits the cell thanks to mitochondrial complementation, which allows damaged mitochondria or mitochondria with altered mtDNA to fuse with healthy mitochondria in order to compensate the deleterious effects of dysfunctional organelles [249].

### Mitophagy and mitochondrial transfer

The quality control of mitochondrial dynamics also includes mitophagy, i.e., a degradation process that removes dysfunctional or damaged mitochondria (**Figure 3D**) [250]. This process is vital to guarantee the physiological functions of cells and tissues and to avoid the onset of diseases like cancer [251]. It also regulates the number of mitochondria in response to the metabolic needs of cells and during some stages of cell development, such as the differentiation of erythrocytes [252].

Mitochondrial removal most often uses the molecular machinery of macro-autophagy, a specific type of autophagy characterized by the formation of mitophagosomes, i.e., double-membrane structures that form vesicles around mitochondria [253, 254]. It can occur either in a selective way or in a non-selective way where autophagosomes sequester mitochondria together with cytosolic components and other organelles [255].

**Figure 4:**
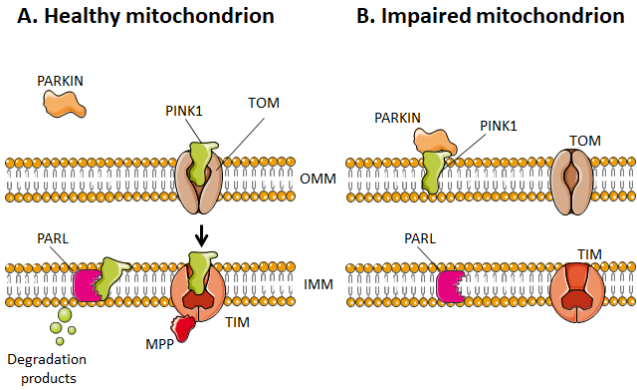
FIGURE 4: Dysfunctional mitochondria are targeted to mitophagy. **(A)** Healthy mitochondria have a polarized outer mitochondrial membrane (OMM), which allows PTEN-induced kinase 1 (PINK1) to cross the membrane and be degraded by presenilin-associated rhomboid-like protein (PARL) at the inner mitochondrial membrane (IMM). **(B)** Impaired mitochondria, instead, have a depolarized OMM, which hinders the entry of PINK1 and, therefore, its degradation. PINK1 can thus bind to parkin to initiate mitophagy. MPP - matrix-processing peptidase; TIM - translocase of inner mitochondrial membrane; TOM - translocase of outer mitochondrial membrane.

Selective mitophagy starts with the evaluation of healthy and damaged mitochondria by PTEN-induced kinase 1 (PINK1) (**Figure 4**). PINK1 is recruited by mitochondria as it contains a mitochondria-targeting sequence [252]. If the mitochondrion is healthy (polarized), PINK1 is transported from the cytosol towards the mitochondrial matrix by translocases of the OMM (TOM) and of the IMM (TIM) (**Figure 4A**). When crossing the IMM membrane, it is cleaved and released back to the cytosol by mitochondrial protease presenilin-associated rhomboid-like protein (PARL) and matrix processing peptidase (MPP) [252, 256]. If, on the contrary, the mitochondrion is dysfunctional, the IMM becomes depolarized, PINK 1 is not cleaved and cannot be transported to the IMS. It accumulates in the OMM (**Figure 4B**). There, it can recruit and activate Parkin by phosphorylating OMM-resident ubiquitins [257, 258] and Parkin itself [259]. Parkin is a cytosolic E3 ubiquitin ligase [260] that, once activated, starts to ubiquitinate proteins in the OMM, including Mfn1, Mfn2, VDAC1, TOM20 and mitochondrial Rho GTPase 1, initializing mitophagy [261–264]. In the cascade, autophagy receptors (such as optineurin) and autophagy initiators (such as unc-51 like autophagy activating kinase 1 [ULK1]) are recruited and activated, as recently detailed in reference [255]. Protein ubiquitination further activates microtubule-associated protein light chain 3 (LC3) [265], which is normally present in the cell cytosol under the form of LC3-I. When activated, LC3 is conjugated to phosphatidylenolamine to form LC3-II [265], which is recruited to autophagosomes during the formation of their double membrane. Besides this canonical pathway, alternatives routes have also been described involving a direct interaction between an activated form of OMM protein FUN14 domain containing 1 (FUNDC1) and LC3, or BH3 only domain proteins (BNIP3 and NIX), beclins and ULK1 [255]. Interestingly, a study by Soubannier *et al.* [266] further showed that mitochondria-derived vesicles can be formed in early oxidative stress response, and these vesicles are directed to lysosomes independently of LC3 as a form of mitophagy complementation.

Dysfunctional mitophagy is associated with tumor initiation and progression in many types of cancers [267, 268]. In response to stresses such as hypoxia and nutrient starvation, mitophagy is activated to reduce the overall mitochondrial mass, thus preserving valuable nutrients and preventing excessive mtROS generation [269, 270]. Even if the exact molecular mechanism is not yet well understood, part of the response could be mediated by receptors that dispose of C-terminal transmembrane domains localized at the OMM, including BNIP3, NIX and FUNDC1 [255]. Parkin has been suspected to be a tumor suppressor: its expression increases oxidative metabolism, limits the Warburg Effect and regulates levels of cyclin D1, cyclin E and cyclin-dependent kinase 4 (CDK4) in cancers [267]. Interestingly, mitophagy appears to have a dual role in cancer treatment resistance, as both its inhibition (in colorectal CSCs) and induction (in preclinical AML models) increased the sensitivity to chemotherapeutic drugs, such as doxorubicin [271]. Mitophagy could also participate in the radioresistance of head and neck cancer cells to γ rays [272].

While mitophagy coupled to mitochondrial biogenesis is a major pathway to maintain a pool of functional mitochondria in cancer cells [273], cancer cells can also acquire intact mitochondria from nonmalignant cells present in the tumor microenvironment. This mitochondrial transfer involves the formation of intercellular tunneling nanotubes (TNTs) and larger microtubes that have been observed in several types of cancer cells [274] based on the initial finding that mitochondria-deficient ρ_0_ cells could acquire mtDNA from host cells [275]. mtDNA transfer was later found to involve full mitochondria transfer between nonmalignant donor cells and malignant receiver cells [276]. In AML, ROS have been identified to drive the formation of TNTs that support full mitochondria exchange between bone marrow stromal cells and AML blasts, which was further shown to contribute to the metabolic capacity of cancer cells [277]. While the molecular mechanisms of TNT formation have been extensively reviewed elsewhere [278], it is in our opinion important to further mention that mitochondrial transfer can also transfer malignant capabilities between cancer cells. In one example, the experimental transfer of mitochondria from an invasive (T24 cells) to a less invasive (RT4 cells) bladder cancer cell line resulted increased invasiveness of RT4 cells [279]. In another example, mtDNA transfer between poorly and highly metastatic lung cancer cells was found to simultaneously transfer the high metastatic traits [280]. In yet another example, Pasquier *et al.* [281] showed that MCF7 breast cancer cells that received mitochondria from endothelial cells became more resistant to doxorubicin chemotherapy. Together, even if TNT formation is extremely difficult to document *in vivo*, these observations support a potential clinical significance of intercellular mitochondrial transfer.

### Mitochondrial dynamics reflect metabolic alterations

Due to frequent fission and fusion events, different forms of mitochondria can be found inside a cell. They can morphologically resemble to small vesicles, short bars or reticular nets, which are a snapshot of shapes resulting from a constantly changing balance. Their organization further depends on cellular types and specific stress conditions [211].

Mitochondrial dynamics have been closely related to the activities that they perform, and changes in mitochondrial morphology have been linked to alterations that occur in cancer [282]. Many studies have demonstrated the existence of a link between energy substrates, oxygen supply and the mitochondrial architecture [283, 284]. Cellular metabolic dysfunctions have been associated with increased mitochondrial fragmentation, whereas hyperfused mitochondria better resist to metabolic insults and, given to the merge, can protect cellular integrity [285]. In β cells of the pancreas, Molina *et al.* [286] have shown that mitochondria that are in a nutrient-rich environment are separated and subject to fission, whereas mitochondria in a starved environment characterized by a severe deficiency of nutrient availability below cellular needs tend to create nets and remain elongated for a long duration. Moreover, starvation induces the accumulation of fatty acid droplets inside mitochondria, shifting cellular metabolism towards FAO for ATP production [287]. Mechanistically, nutrient starvation induces mitochondrial elongation through cAMP-activated PKA that inhibits mitochondrial fission and protects the organelles against autophagosomal degradation [288, 289]. In cancer, existing data suggest that a hyperfused state of mitochondria supports the survival of cancer cells not only by maintaining the production of ATP, but also by compensating for damaged mitochondria, sustaining intramitochondrial exchanges of fatty acids and avoiding metabolic reprogramming towards autophagy [287–289].

## THERAPEUTIC STRATEGIES TARGETING MITOCHONDRIA IN CANCER CELLS

Given the key functions that mitochondria exert in cancer cells, several strategies have been imagined and tested that considered mitochondria as anticancer targets (**Table 1**). We here offer a brief overview of major approaches aiming to modulate mitochondrial anaplerosis, mitochon-drial turnover, the TCA cycle, the ETC, mtROS and mitochondria-driven apoptosis.

**TABLE 1.** Therapeutic strategies targeting mitochondria in cancer with clinical perspectives.

**Compound name**	**Targeted functions**	**Molecular targets**	**Phase**	**ClinicalTrials.gov identifier or reference**
2-Deoxy-*D*-glucose	Mitochondrial anaplerosis	GLUTs and HKs	Phase I	NCT00096707
			Phase III	[302, 303]
3-Bromopyruvate	Mitochondrial anaplerosis	HK2 and GAPDH	Case study	[307, 308]
Etomoxir	Fatty acid oxidation	Carnitine palmitoyltransferase I	Preclinical	[314, 315]
Mdivi-1/Dynasore	Mitochondrial turnover	DRP1	Preclinical	[368–371]
CB-839	TCA cycle	Glutaminase	Phase I	NCT02071927, NCT02071888
CPI-613	TCA cycle	Pyruvate dehydrogenase and α-ketoglutarate dehydrogenase	Phase I	NCT02168140, NCT02232152
			Phase I/II	NCT01766219
AG-221	TCA cycle	Mutant IDH2-R140 and IDH2-R172	Phase I/II	NCT01915498, NCT02273739
AG-881	TCA cycle	Mutant IDH1/2	Phase I/II	NCT02492737, NCT02481154
Carboxyamidotriazole	ETC	Complex I	Preclinical	[319]
Fenofibrate	ETC	Complex I	Preclinical	[320]
Metformin	ETC	Complex I	Phase III	NCT01101438
Papaverin	ETC	Complex I	Phase I	NCT03824327
Lonidamine*	ETC	Complex II	Phase II	NCT00237536
			Phase III	NCT00435448
Atovaquone	ETC	Complex III	Phase I	NCT02628080
Arsenic trioxide	ETC	Complex IV	Preclinical	[348–350]
mitoTEMPO	ROS signaling	Superoxide	Preclinical	[89]
MitoQ	ROS signaling	Superoxide	Preclinical	[354]
Photodynamic therapy	Mitochondria-driven apoptosis	Cytochrome *c* release	Phase I	NCT03053635
			Phase II	NCT03945162
Curcumin	Mitochondria-driven apoptosis	Cytochrome *c* release	Phase III	NCT02064673,
			Phase II	NCT02944578, NCT02782949
Aloe-emodin	Mitochondria-driven apoptosis	Cytochrome *c* release	Preclinical	[362]
Betulin	Mitochondria-driven apoptosis	Cytochrome *c* release	Preclinical	[363]
Resveratrol	Antioxidant modulators	Cytochrome *c* release	Phase I	NCT00256334, NCT00433576
α-tocopheryl succinate (α-TOS)	Mitochondrial destabilization	GSTP1-1 and GSTO1-1	Preclinical	[377]
Canfosfamide (TLK286)	DNA replication	Pro-drug bio-activated by GSTP1-1 in an alkylating agent	Phase III	NCT00102973
Brostallicin	DNA replication	Pro-drug activated by GSTP and GSTM	Phase II	NCT00060203, NCT01091454
Ketogenic diet	Glycolysis	Mitochondria in cancer cells that would not use ketone bodies as a fuel	Pilot	NCT01535911
			Not applicable	NCT03075514, NCT02286167, NCT01754350, NCT03278249
			Phase I	NCT00575146, NCT03451799, NCT01865162
			Phase I/II	NCT02046187, NCT02939378
			Phase II	NCT02302235

* withdrawn from clinical studies. DRP1 - dynamin-related protein 1; ETC - electron transport chain; GLUT - glucose transporter; GST - glutathione S-transferase; HK - hexokinase; IDH - isocitrate dehydrogenase; ROS - reactive oxygen species; TCA - tricarboxylic acid (cycle).

### Targeting mitochondrial anaplerosis

Targeting pathways supporting mitochondrial anaplerosis, i.e., glycolysis, glutaminolysis and FAO, has been considered as an anticancer approach. While these anaplerotic pathways may in part support cancer cell survival and proliferation independently of mitochondrial metabolism, their inhibition results in TCA cycle fuel deprivation.

2-Deoxy-*D*-glucose (2DG) is a competitor of glucose for glucose transporters (GLUTs) and HKs, which are often overexpressed in cancer cells, offering some anticancer selectivity for the treatment [290]. By interfering with glucose uptake and phosphorylation, 2DG reduces the availability of pyruvate for mitochondria, thus impairing mitochondrial anaplerosis and OXPHOS. In addition to its anti-metabolic activity, preclinical studies reported that 2DG exerts additional anticancer effects that comprise an anti-angiogenic activity [291], inhibition of cancer metastasis [292] and inhibition of the viral replication of Kaposi's sarcoma-associated herpes virus [293]. It can also improve the effects of autophagy inhibition [294–296]. However, clinical trials were generally inconclusive, as 2DG as a standalone treatment did not show significant anticancer activity at tolerated doses for patients [297]. Additional *in vitro* and *in vivo* preclinical studies nevertheless suggested its possible application in combination with conventional chemotherapy, including cisplatin [298, 299] and doxorubicin [300, 301]. Promising results have also been obtained in combination with radiotherapy for the treatment of glioblastoma, and a Phase III clinical is currently ongoing [302, 303].

3-Bromopyruvate (3BP) is an alkylating agent that, among other effects, has been reported to inhibit glycolysis. 3BP indeed inhibits HK2 [304] and GAPDH [305] among a larger list of targets [306]. However, even if promising case studies have been published supporting that 3BP exerts anticancer effects in humans [307, 308], to our knowledge no clinical trial has been completed to date. Although a promising compound, 3BP faces several limitations for its clinical utility, including a rapid deactivation by GSH and a burning sensation when administered intravenously. In order to limit side effects and potentiate its anticancer properties, 3BP can be formulated in liposomes, PEG-liposomes or other targeted or untargeted nanocarriers [309].

Glutaminolysis has been shown to support cancer cells growth, in particular in human pancreatic ductal adenocarcinoma (PDAC) [310, 311] and triple negative breast cancer [312]. Glutaminase 1 (GLS1) has been proposed as a target for anticancer treatment, however there are no pharmacological treatments available today.

Targeting FAO is another potential anticancer strategy. A special focus has been set on carnitine palmitoyltransferase I (CPT1), an enzyme located at the OMM that converts acyl-CoA (the end product of FAO) to acyl-carnitine that crosses mitochondrial membranes in order to fuel the TCA cycle in the mitochondrial matrix [313]. The irreversible CPT1 inhibitor etomoxir has been shown to improve the effectiveness of radiotherapy [314], and it may be used to treat cachexia, a syndrome associated to elevated FAO in cancer patients [315]. Etomoxir has been tested in clinical trials for type 2 diabetes and congestive heart failure, which revealed a safe to use profile of the drug [316, 317]. However, to our knowledge, no clinical trial in cancer patients has been initiated today.

### Targeting the TCA cycle

Whether and how to selectively target the TCA cycle in cancer cells has been extensively explored in the past years and recently reviewed in details by Anderson *et al.* [35]. Importantly, several Phase I and II clinical trials have been conducted involving drugs capable of inhibiting deregulated pathways related to the TCA cycle. They include CB-839, a specific inhibitor of glutaminase (NCT02071927 and NCT02071888); CPI-613, a lipoate analog inhibiting pyruvate dehydrogenase and α-KG dehydrogenase that was recently tested in Phase I and II clinical trials as a single agent or in combination with standard chemotherapy to treat diverse types of cancers (NCT02168140, NCT02232152 and NCT01766219); and enasidinib/AG-221, an orally available inhibitor of mutant IDH2-R140 and IDH2-R172 that undergoes Phase I/II clinical trials as a single agent for the treatment of AML, angio-immunoblastic T-cell lymphoma and glioma (NCT01915498 and NCT02273739). In addition, AG-881 is a promising orally available dual inhibitor of mutant IDH1 and mutant IDH2 that was in Phase I/II clinical trial until this year, recruiting AML patients with mutant IDH1/2, as well as glioma patients (NCT02492737 and NCT02481154).

### Targeting the ETC

As reviewed in the section ‘Antioxidant defenses in mitochondria‘, cancer cell mitochondria can also display alterations in the functions of ETC complexes that constitute attractive anticancer targets.

#### Complex I

A multiplicity of compounds have been tested *in vitro* and *in vivo* to inhibit Complex I [318], which include carboxyamidotriazole [319] and fenofibrate [320]. However, the most advanced drugs targeting Complex I are biguanides metformin and phenformin. These two compounds are FDA-approved drugs widely used for diabetes treatment [321]. Interestingly, they can also impair the proliferation of several cancer cell lines [322, 323], and *in vivo* studies further demonstrated that metformin and phenformin inhibit tumor growth and metastasis formation in several animal models [324–328]. While these two drugs were believed to primarily act through AMPK activation, two studies published in the year 2000 demonstrated that metformin primarily inhibits ETC Complex I in cancer cells, consequently inhibiting OXPHOS and activating AMPK [329, 330]. The mode of action of these biguanides involves inhibition of ubiquinone reduction [331]. This finding opened new perspectives for the use of biguanides in the field of cancer. In fact, metformin is currently tested in several clinical trials for cancer patients [332]. Until now, most promising results have been obtained in Phase III clinical trial NCT01101438, which tested the efficacy of metformin for the treatment of breast cancer, other nonmetastatic cancers and cancers with a smaller degree of malignity [333]. After receiving metformin twice a day for five years after diagnosis, patients experienced a significant improvement of progression-free survival. Papaverin, a non-narcotic opioid usually used for the treatment of vasospasms and erectile dysfunction by inhibiting phosphodiesterase 10A [334], has also been reported as a Complex I inhibitor in cancer cells [335]. As a consequence, Benej *et al.* [335] reported that papaverin can, through oxygen sparing, radiosensitize lung and breast cancer cells and tumors *in vivo*. With the additional description of anticancer effects of papaverin on prostate [336], breast [337] and hepatocarcinoma [338] cancer cells, as well as on glioma xenografts in mice [339], a Phase I clinical trial is ongoing where papaverin hydrochloride is tested as a radiosensitizer in non-small cell lung cancer patients (NCT03824327).

#### Complex II

Comparatively to Complex I, there are currently no well characterized Complex II inhibitors. Lonidamine has recently been shown to inhibit Complex II in isolated mitochondria and in DB-1 melanoma cells [340, 341]. However, the compound did not demonstrate any benefit in two randomized Phase III clinical trials in combination with chemotherapy, and was therefore withdrawn from clinical studies [340].

#### Complex III

Atovaquone is an FDA-approved drug used to treat pneumocystis, pneumonia and malaria [342]. This drug is a ubiquinone analogue that acts as a Complex III inhibitor in parasites, cancer cell lines and breast CSCs, diminishing the oxygen consumption rate and reducing tumor hypoxia at pharmacologically acceptable concentrations [343–347]. Atovaquone is currently tested in a Phase I clinical trial (NCT02628080) for its effects on hypoxia in non-small cell lung carcinoma in a pre-operative window of opportunity study. While recruitment has been completed, no results have been published yet regarding this trial.

#### Complex IV

Arsenic trioxide has been described as a Complex IV inhibitor and is FDA-approved for the treatment of acute pro-myelocytic leukemia. In the past years, it has been explored in other types of cancer. Preclinically, because it inhibits cell respiration, arsenic trioxide was shown to decrease hypoxia in Lewis lung carcinoma and transplantable mouse liver tumors, leading to an improvement of the response of mice to radiotherapy [348]. Nitric oxide and hydrocortisone are other compounds that, among other effects, can inhibit Complex IV and increase the efficacy of radiotherapy preclinically [349, 350].

#### Complex V

To our knowledge, no studies involving *in vivo* or clinical trials have been reported with drugs targeting ATP synthase in cancer models.

### Targeting mitochondrial ROS production

The use of mitochondria-targeted antioxidants like mitoTEMPO and mitoQ [89] has proven to be a good strategy to repress the migratory, invasive and metastatic phenotypes of cancer cells. Both drugs repress mtROS-induced activation of the TGFβ pathway [89]. MitoTEMPO is a superoxide scavenger able to mimic mitochondrial SODs [351]. It can easily pass through lipid bilayers and selectively accumulates in negatively charged mitochondria thanks to a positively charged triphenylphosphonium moiety. MitoQ is a very similar compound, with an ubiquinone covalently bound to triphenylphosphonium. Similar to mitoTEMPO, it can rapidly cross biological membranes and concentrate up to 100-fold in mitochondria [352]. It can access the membrane core of mitochondria, acting as a chain-breaking antioxidant, which further allows the recycling of MitoQ to its ubiquinol form via reduction by ETC Complex II [353]. Besides its antimetastatic potential, MitoQ as a mitochondria-targeted antioxidant can successfully decrease KRAS-induced pancreatic tumorigenesis *in vivo* [354].

### Restoring mitochondria-driven apoptosis

Strategies that stimulate cytochrome *c* release can be used to induce apoptotic cancer cell death. Several studies [355–357] have indeed proposed that photodynamic therapy (PDT) can induce damage in cancer cells by disturbing the mitochondrial membrane potential, thus triggering the release of cytochrome *c* and activating caspase-dependent cell death. Several photosensitizers used in PDT have been approved for clinical applications or are under clinical trials [358].

Additionally, the use of natural compounds to induce cancer cell apoptosis has shown interesting results. For examples, resveratrol [359, 360], curcumin [361], aloe-emodin [362] and betulin [363] were shown to stimulate apoptosis in various cancer cell lines by increasing cytochrome *c* release from mitochondria. Resveratrol is tested in Phase I clinical trials with colon cancer patients (NCT00256334, NCT00433576). Encouraging preclinical results obtained with FDA-approved curcumin in cancer were an incentive to launch several clinical trials in cancer patients (NCT02064673, NCT02944578, NCT02782949). The non-toxicity towards non-cancer cells demonstrated by these natural compounds added to the low toxicity induced in normal cells by PDT suggests that targeting cancer cells by restoring cytochrome *c*-driven apoptosis deserves further research efforts.

### Targeting mitochondrial turnover

Mitochondrial fission, fusion and mitophagy have been investigated as potential anticancer targets. However, few studies specifically investigated mitophagy compared to more general autophagy.

Mdivi-1 can block fission by inhibiting the GTPase activity of DRP1 [364], thus preventing mitophagy. For treatments longer than 24 h, mdivi-1 has a cytostatic effect [365], which may be an additional advantage over mitochondrial impairment [366] in cancer therapy. Dynasore is another GTPase inhibitor of DRP1 [367]. Inhibition of mitochondrial fission by dynasore has been shown to suppress cancer cell proliferation and to induces apoptosis in A549 lung cancer cells [368]. It inhibits migration and/or invasion in different cancer cell lines, including bladder cancer cell line T24 [369], lung cancer cell line H1080 [370] and osteosarcoma cancer cell lines MNNG/HOS [371], MG-63 [371], and U2OS [370]. *In vivo* in mice, dynasore showed additive effects when combined to cisplatin [371].

Inducing mitochondrial fusion can also have anticancer effects, as illustrated by S3, a small natural molecule that has been shown to promote fusion hence inhibiting mitophagy [372]. Its mechanisms of action remain to be elucidated, which will necessitate *in vivo* studies. Of note, inhibiting mitophagy may be one of the mechanisms of action of Temsirolimus, a mTOR inhibitor already in use for other clinical applications than cancer [373, 374].

### Other mitochondrial modulators

In addition to mitochondrial inhibitors, other compounds can indirectly modulate mitochondrial functions. Among them is α-tocopheryl succinate (α-TOS), an analogue of α-tocopherol (vitamin E). α-tocopherol is a potent inhibitor of cytosolic GSH S-transferases GSTP1-1 [375] and GSTO1-1 [376], i.e., enzymes that normally detoxify endogenous and exogenous compounds by catalyzing the conjugation of electrophilic centers to GSH. Its analogue α-TOS selectively induces apoptosis in cancer cells by destabilizing mitochondria [377]. This activity, however, seems to be independent of GST inhibition, and would rather primarily involve ETC Complex I [378] or Complex II [379] inhibition.

Interestingly, the catalytic activity of GSTs could also be used to bio-activate prodrugs, allowing their selective accumulation in cancer cells with high expression of some GST isoenzymes. For example, canfosfamide/TLK286, a modified GSH analogue and nitrogen mustard prodrug, is bio-activated by GSTP1-1 in an alkylating metabolite capable of covalently binding DNA [380]. This compound reached Phase III clinical trials with good tolerability [381–383]. Brostallicin is another example of a GST-activated prodrug [384–386]. It is currently in Phase II clinical trials.

Ketogenic diets (K.Ds) based on a high fat and low carbohydrate alimentation have the objective to limit glucose availability for tumors [387]. They were initially developed as a treatment for rare metabolic diseases, such as GLUT1 and pyruvate dehydrogenase (PDH) complex deficiencies [388, 389]. KDs have been reported to decrease blood glucose levels and to increase ketone body use, leading to a shift from glycolysis to respiration. Their anticancer activity is based on the assumption that cancer cells with altered mitochondria should not be able to use ketone bodies [390, 391]. Several studies associating mitochondrial modulators to KDs have been performed to treat cancer patients, which have recently been reviewed by Weber *et al.* [387]. The most significant findings pertain to case reports of patients with glioblastoma [392–394]. While anticancer activities have been reported, many clinical trials are still ongoing. The results of these studies are expected to confirm whether KDs, and which type of KD, could be used as a nutritional support to improve the outcome of some types of cancers.

## CONCLUSIONS

Introductory statements in the scientific literature too often describe cancer cells as being constitutively glycolytic. This reductionist view is based on the rediscovery of the Warburg effect that has strongly increased our knowledge of cancer biochemistry since the year 2000, with the drawback that studying other metabolic pathways has been under pursued for almost a decade. If indeed the Warburg phenotype provides a biosynthetic advantage for cancer cell proliferation [143, 395, 396], it is evident that not all cancer cells simultaneously proliferate in experimental and clinical tumors. Hence, tumor metabolism is generally characterized by the same heterogeneity as the phenotypic heterogeneity of the cancer cells that compose the tumor. Good examples are the metabolic cooperativity that exists between oxidative and glycolytic cancer cells in many tumor types [397, 398] and the observation that, in a given tumor, metastatic progenitor cells [89] and CSCs [87] have a different metabolic behavior than the bulk population of cancer cells. It is nevertheless noticeable that mutations in genes encoding TCA cycle enzymes [111, 121, 399] or ETC subunits [67, 105, 106] logically limit this metabolic plasticity, but they are rare events in rare tumor types.

This review paper aimed to contribute to re-center mitochondria in the overall metabolic map of cancers. We therefore attempted to provide a comprehensive overview of the many functions that these organelles exert in cancer cells, not only as powerhouses, but also as dynamic signaling organelles controlling cell survival and death, motility, stemness and resistance to treatment. However, key questions have not yet been answered by the scientific community. In our opinion, a significant issue is to determine whether metabolic alterations are a cause or a consequence of the malignant process. Elements in the literature support both possibilities. For example, it is clear that anaerobic glycolysis coupled to lactic fermentation [170, 172, 400] and increased mitophagy [401] are adaptive survival pathways to hypoxia, hence consequences of hypoxia. The situation is less clear for aerobic glycolysis, where an allosteric control of pyruvate kinase M2 by fructose-1,6-bisphosphate (that activates the enzyme) and alanine (that inhibits the enzyme) may dictate progression in the cell cycle [402], or, vice versa, the cell cycle could impose metabolic cycles to cancer cells. These cycles would be characterized by alternations of energy production and biosynthesis. For mitochondria in particular, experiments of mitochondrial transfer have clearly demonstrated that these organelles can carry and transfer malignant information, such as the capacity to metastasize [280]. Identifying whether specific metabolic alterations drive or merely follow the phenotypic evolution of cancer cells is not trivial, as in the first case targeting these changes may block phenotypic progression, whereas in the second case metabolic plasticity could rapidly overcome therapeutic interventions.

In the last section of this review, we briefly described the most advanced therapeutic compounds targeting mitochondria in cancer. For experts in the field, it is obvious that the list is short and that most of the drugs do not have unique targets. It probably reflects that the field is still in its infancy, in the sense that several important fundamental discoveries are still to be made that would identify precise mitochondrial alterations in cancer allowing specific anticancer interventions. In other words, the molecular definition of ‘oncogenic mitochondria', i.e., mitochondria that carry and can transfer malignant information, should be a priority for basic research. Because they carry their own genetic material and are subjected to environmental changes within cells (such as pH, pO_2_, the availability of metabolites and exposure to treatments) and as metabolic sensors of the extracellular microenvironment, it is possible that mitochondria could undergo Darwinian selection during tumor progression. In support of this hypothesis, several papers already identified mutations in mtDNA with functional effects [403], for example in renal oncocytomas [404] and pancreatic cancers [405]. The development of mtDNA editing tools [406] is expected to provide experimental strategies to track, characterize and repair oncogenic mitochondria, which will further require a deep understanding of mitochondrial epigenetics in cancer.

## Abbreviatons:

2DG: – 2-deoxy-D-glucose;
2HG: – D-2-hydroxyglutarate;
3BP: – 3-bromopyruvate;
α-KG: – α-ketoglutarate;
α-TOS: – α-tocopheryl succinate;
ACO2: – aconitase 2;
AML: – acute myeloid leukemia;
AMPK: – AMP kinase;
Atg: – autophagy-related;
Bcl-2: – B-cell lymphoma 2;
CoQ: – coenzyme Q;
CS: – citrate synthase;
CSC: – cancer stem cell;
DRP1: – dynamin-related protein 1;
EMT: – epithelial to mesenchymal transition;
EOC: – epithelial ovarian carcinoma;
ER: – endoplasmic reticulum;
ETC: – electron transport chain;
FAO: – fatty acid oxidation;
FH: – fumarate hydratase;
FoxO1: – forkhead O family protein 1;
FUNDC1: – FUN14 domain-containing 1;
GAPDH: – glyceraldehyde-3-phosphate dehydrogenase;
GLUT: – glucose transporter;
GSH: – glutathione;
GST: – glutathione S-transferase;
HIF: – hypoxia-inducible factor;
HK: – hexokinase;
Hsp70: – heat shock protein 70;
IDH: – isocitrate dehydrogenase;
IMM: – inner mitochondrial membrane;
IMS: – intermembrane space;
KD: – ketogenic diet;
KRAS: – V-Ki-ras2 Kirsten rat sarcoma viral oncogene homolog;
LC3: – microtubule-associated protein light chain 3;
LDH-1: – lactate dehydrogenase-1;
MAVS: – mitochondrial anti-viral signaling;
MCL-1: – myeloid leukemia cell differentiation protein-1;
Mdivi-1: – mitochondrial division inhibitor-1;
Mff: – mitochondrial fission factor;
Mfn: – mitofusin;
mtDNA: – mitochondrial DNA;
mtROS: – mitochondrial ROS;
mtSNP: – single nucleotide polymorphism in mtDNA;
Nrf2: – nuclear factor erythroid-derived 2-like 2;
OMM: – outer mitochondrial membrane;
Opa1: – optic atrophy protein 1;
OXPHOS: – oxidative phosphorylation;
PDT: – photodynamic therapy;
PEP: – phosphoenolpyruvate;
PKA: – cAMP-activated protein kinase A;
PINK1: – phosphatase and tensin homolog-induced kinase 1;
PTEN: – phosphatase and tensin homolog;
ROS: – reactive oxygen species;
SCO2: – synthesis of cytochrome oxidase 2;
SDH: – succinate dehydrogenase;
Sirt: – sirtuin;
SOD: – superoxide dismutase;
TCA: – tricarboxylic acid (cycle);
TERT: – telomerase reverse transcriptase;
TGFβ: – transforming growth factor β;
TIGAR: – TP53-induced glycolysis and apoptosis regulator;
TNT: – tunneling nanotube;
VDAC: – voltage-dependent anion channel.
